# Effects of teach-back health education (TBHE) based on WeChat mini-programs in preventing falls at home for urban older adults in China: a randomized controlled trial

**DOI:** 10.1186/s12877-022-03297-9

**Published:** 2022-07-23

**Authors:** Qiong Ye, Yuting Yang, Miao Yao, Yongwei Yang, Ting Lin

**Affiliations:** 1 The School of Nursing, Fujian Medical University, Fuzhou, Fujian China; 2 Ningde Municipal Hospital of Ningde Normal University, Ningde, Fujian China

**Keywords:** Accidental falls, Application software, Community-dwelling elderly, Health promotion, Internet, Randomized controlled trial

## Abstract

**Background:**

Falls are common among adults aged 60 years and older because of physiological changes. Most falls in older adults occur most often at home. Coupled with the lack of awareness and knowledge of preventing falls, the proportion of injuries and deaths among older adults due to falls is increasing yearly. Our study developed a WeChat mini-program for urban elderly to implement teach-back health education (TBHE) that a repeated cycle process of health education, assessment, and re-education in preventing falls at home.

**Objectives:**

This study aimed to evaluate the application effect of the TBHE-based WeChat mini-program on health education knowledge for fall prevention at home for urban older adults.

**Design:**

A single-blinded, two-arm parallel-group, randomized controlled trial was conducted.

**Setting:**

Three residential communities, named Hot Spring Apartment, Hualinyuan, and Dongtang Community in Gulou District, Fuzhou, China.

**Participants:**

Participants were older adults recruited from communities in Fuzhou from January to March 2021.

**Methods:**

Fifty-nine participants agreed to participate and were assigned randomly to the intervention group (*n* = 29) or the control group receiving traditional health education (*n* = 30). Each participant in the intervention group received twice a week for a total of 8 weeks of health education interventions performed by the first author that she is intervenor according to specific themes. The trial statistician, recruiters, and participants were blinded to group allocation. The intervenor (first author) was blinded to the study hypotheses. To evaluate the effects of the intervention, we assessed participants’ knowledge total score and scores of physiology and disease; drug application; mental, cognitive, and spiritual well-being; lifestyle; and house environment at baseline and 1-week post-intervention and compared scores between two groups. A two-way repeated-measures analysis of variance was conducted to examine the effects of time, group, and their interaction.

**Results:**

There was a significant difference in knowledge of house environment (*p* = 0.003) between the two groups. Within groups, total and five dimensions knowledge scores had a significant difference (*p* < 0.001). Moreover, interaction effects were significant on drug application (*p* = 0.012) and mental, cognitive, and spiritual well-being (*p* = 0.015).

**Conclusions:**

The TBHE can improve knowledge on fall prevention at home among urban older adults. The TBHE based on the WeChat mini-program could enhance the efficiency and effectiveness of being educated among urban older adults.

**Trial registration:**

Chinese Clinical Trial Register: ChiCTR2100052946; reg date: 06/11/2021.

**Supplementary Information:**

The online version contains supplementary material available at 10.1186/s12877-022-03297-9.

## Background

China has the largest elderly population in the world. The seventh census [1] showed that at the beginning of November 2020, the number of people aged 60 years and above in China was 264 million, accounting for 18.70% of the total population. Falls are common among adults aged 60 years and above due to physiological changes [2, 3]. Thus, the incidence of falls increases with age. Approximately one-third of individuals above 65 years fall each year, and the incidence of falls in persons above 80 years is as high as 50% [4]. In addition, 71.20% of older adults will experience contusion, abrasion, sprain, fracture, and even death after falling [5]. Falls have become the leading cause of injury among older adults and the second leading cause of death due to injury in older adults [6]. The most common place where falls occur for older adults is at home. Jiayuan [7] conducted a survey on the status of falls among 561 older adults in urban communities and found that falls were more likely to occur at home than anywhere else, accounting for 45.6%. Tolulope [8] reviewed and analyzed the case data of elderly patients in the U.S. National Trauma Database from 2003 to 2006 and found that more than 42% of older adults fell at home. Therefore, to reduce the overall incidence of falls in the elderly, it is required to start by preventing falls at home.

According to the China Health and Retirement Longitudinal Study [9] that screened 4736 eligible older adults, the incidence of falls in rural areas (22.00%) is higher than that of urban areas (18.70%), even the incidence of severe falls in both urban and rural areas are comparable. Rural older adults are engaged in long-term agricultural work and have a low awareness of fall prevention [10]. Agriculture-based work methods can easily lead to the rapid degeneration of musculoskeletal system functions, increasing the risk of falls [9]. Therefore, our research focuses on urban seniors who are less affected by physiological changes, hoping to be inspired by the practice of guiding urban older adults to prevent falls. Then, we could scale it up to rural older adults who also need guidance on fall prevention.

Health education is effective for fall prevention at home for older adults [11] and has received extensive attention due to its cost-effectiveness, simplicity, and ease of implementation [12]. Minglong [13] selected 300 elderly community residents and randomly divided them into two groups, which received one-to-one fall prevention health education and traditional centralized education for 6 months, respectively. The former can significantly improve older adults’ fall prevention knowledge, attitudes, and behaviors, thereby reducing the incidence and adverse consequences of falls. Generally, health education is an effective method to improve older adults’ knowledge on fall prevention at home; however, it is a one-way health education process without real-time feedback through which information is transmitted from medical service providers to patients [14]. Therefore, its effect cannot be effectively guaranteed, and medical service providers may not fully understand the degree of knowledge mastery of the individuals educated.

Teach-back is a communication technique in which patients are asked to repeat or describe what they understand about their condition, treatment, or any instructions given during treatment to confirm their understanding [15]. Patient health education based on teach-back has achieved satisfactory results, which could help patients solve possibly existing health literacy issues [16, 17]. If health providers appropriately utilize teach-back, they can check if their instructions are being delivered correctly. And patients can have the opportunity to demonstrate their understanding of the instructions given or specific treatment skills necessary for their conditions [15]. Therefore, teach-back health education can evaluate the teaching effect in real-time and multiple times and strengthen the health education content according to the evaluation results to achieve the desired effect.

With the rapid development of information technology in China, WeChat, an emerging social media, is preferred by the public because of its convenience and personalization [18]. WeChat is a free mobile application launched by Tencent in January 2011 with multimedia information and communication as its core function [19]. WeChat has rich functions that could achieve the purpose of expanding social interaction, maintaining social relations, and meeting the demands of expressing emotions [18]. Users could quickly send pictures, texts, voice notes, and videos across operators and platforms through mobile phones, tablets, or web pages, conduct single or group chats with others, share content through Moment with friends, and receive news pasted by public platforms [18].

Simultaneously, the scale of older netizens is constantly expanding. Since 2011, the proportion of netizens aged 60 years and above in China has increased from 2.40 to 11.50%, and the growing scale of urban elderly netizens far exceeds that of rural areas [20, 21]. The number of urban older netizens using WeChat is also increasing. Tencent Research Institute [22] conducted a household survey from January to June 2018 and found that 49.60% of 1399 individuals aged above 55 years in China were using social media, and their most common social media was WeChat. There are few studies in China on fall prevention health education among older adults based on WeChat. Mengchao [23] conducted a randomized controlled trial in which WeChat-based fall education was administered to 160 hospitalized elderly patients. This study found that this intervention reduced fall incidence among older patients and improved their basic knowledge on fall prevention and cognition. Although WeChat education improved fall prevention knowledge among elderly hospitalized patients, the study [23] also had limitations. For example, after Mengchao [23] using the Morse Fall Scale (MFS) to assess patients’ risk of falls, researchers invited the patients at risk of falls to participate in their study. Therefore, it is uncertain whether their results are applicable to older adults who are also at risk of falls at home. In addition, the memory, comprehension, and reaction ability of older adults may decline to various degrees with the increase in age [24]. One-way and one-time health education without real-time feedback may not achieve effective long-term effects [14].

Fall incidence at home is still high among urban older adults [7, 9], and falls lead to different degrees of harm in this population [5]. Health education is a significant strategy to reduce fall incidence at home because it could promote fall prevention and cognition [13]. With information technology development, traditional health education can no longer meet the demands of older adults, and information-based health education is becoming a research hotspot [14]. Moreover, because traditional health education without real-time feedback is only a one-way process in which information is transmitted from medical service providers to patients, its effect cannot be effectively guaranteed [14]. Teach-back health education allows patients to continuously receive correct instructions or specific skills from healthcare providers to improve their health literacy [15]. However, there are no reports on studies that administered teach-back health education to urban older adults to prevent falls at home.

This study aimed to explore the application effect of teach-back health education (TBHE) based on the WeChat mini-program in preventing falls at home for urban older adults. Before conducting this study, we first developed a TBHE WeChat mini-program that is easy to operate for older adults. It was prepared as an internet platform for online health education and has passed pre-experiment. Our study is registered in the Chinese Clinical Trial Register (ChiCTR2100052946) as a single-blind, two-arm, parallel-group, randomized controlled trial on November 6, 2021. It is the first study on TBHE for improving fall prevention knowledge at home among urban older adults using a WeChat mini-program. Our hypothesis was that the TBHE could improve knowledge on fall prevention at home among urban older adults. The TBHE based on the WeChat mini-program could enhance the efficiency and effectiveness of being educated among urban older adults.

## Methods

### Feasibility study

Ten urban older adults who met the inclusion criteria were invited by convenience sampling to participate in a pre-experiment on the feasibility of the WeChat mini-program from February 22 to March 1, 2021. These individuals participated only in the pre-experiment. The researchers informed the participants about the purpose of testing the WeChat mini-program. After obtaining their consent, they started using the WeChat mini-program for testing. All participants experienced a smooth process and were able to complete each module of the WeChat mini-program under guidance. The older adults stated that the WeChat mini-program had an appropriate font size and was easy to use and operate, and also that it was easy to watch videos in it. Their experience satisfaction was 100%. The WeChat mini-program video played smoothly and did not freeze.

### Participants

First, a simple random sampling was used to select one urban area named Gulou District in Fuzhou, China. Then, three communities were randomly selected from all residential communities in Gulou District, including Hot Spring Apartment, Hualinyuan, and Dongtang Communities. Further, older adults in these three communities who met the inclusion criteria were invited to participate in the present study. The intervention lasted for 8 weeks, twice a week, from March 9 to April 30, 2021.

Inclusion criteria included age older than 60-year-old, living in an urban community for 6 months or above, possessing a smartphone and can use WeChat, ability to write and understand simple Chinese characters, and signing the written informed consent. Exclusion criteria were hearing impairment, speech impairment, severe cognitive impairment, mental illness, other severe or terminal diseases, and currently participating in a study related to similar health education interventions. The determination of each item of exclusion criteria was based on the researchers’ judgment. Researchers would ask the following questions when touching older adults: (1) Are you currently taking certain drugs? (2) Do you currently have diagnosed diseases? (3) Are you currently participating in some scientific research projects? If so, could you tell us the specific project theme? Through these questions, the researchers judged whether older adults had normal hearing and speech expression ability, whether they had severe diseases, were participating in certain scientific research projects, and were suitable for participating in this study.

In this study, sample size was calculated using the formula for two samples mean comparison in a randomized controlled trial. Assuming a power of 0.9, alpha of 0.05, and standard deviation of change in knowledge level of 50%, each study arm required at least 27 participants. Assuming a drop-out rate of 10%, 30 participants were invited in each group. This study recruited 60 participants, including 30 participants in the intervention group and 30 in the control group from January 23 to March 4, 2021.

### Recruitment, randomization, blinding, and treatment allocation

**Fig. 1:**
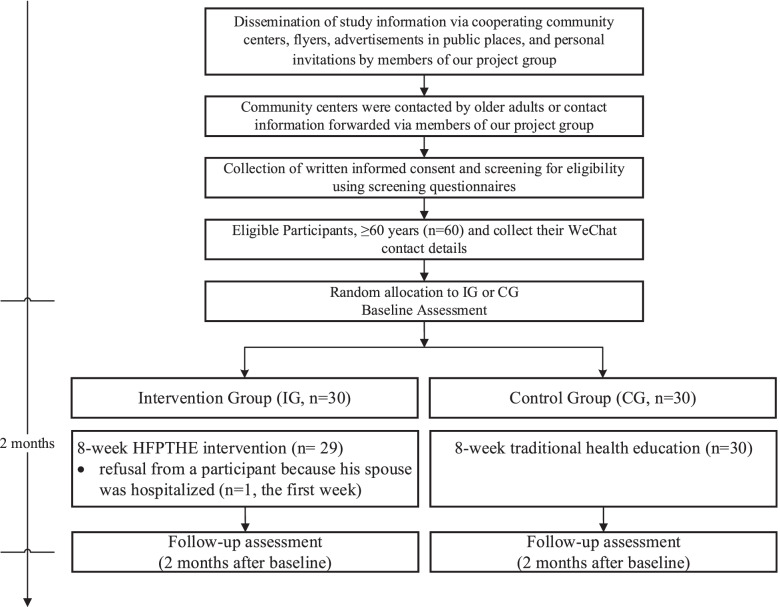
Overview of the recruitment and study timeline

Participants were recruited through cooperating community centers, flyers, and advertisements in public places (parks or squares) and via personal invitations by two members of our team dedicated to recruitment. Participants were randomized into the intervention group or the control group through a third party using a random sequence set generated from the Research Randomizer Website (https://www.randomizer.org/). The sequence of numbers contained 60 non-repeating numbers ranging from 1 to 60. Randomized numbers were put in two sequentially numbered, sealed, and opaque envelopes numbered 1 and 2 by a third party to ensure allocation concealment. Envelope 1 was the intervention group, and envelope 2 was the control group. After the research assistants recruited 60 older adults according to the inclusion criteria, participants were numbered from 1 to 60 and grouped according to numbers from envelopes. Recruitment research assistants were blinded in the trial design and group allocation, and the trial statistician was blinded to group allocation. Participants were blinded to group allocation, although they were aware they were in a study that teach-back health education (TBHE) based on WeChat mini-programs to prevent falls at home. Due to the nature of this study, the intervenor (the first author, FA) could not be blinded to the group allocation but blinded to the study hypotheses and receive the names of participants who accepted The TBHE intervention. Then, FA established two WeChat groups. In the WeChat group, FA explained the purpose of forming the WeChat group and what members needed to do to gain the trust and cooperation of older adults. An overview of the recruitment and study timeline is shown in Fig. 1.

### Intervention

#### Intervenor

The First author, a supervisor nurse with 10 years of clinical nursing work and health education experience, carried out the health education intervention. She is familiar with knowledge on fall prevention at home and has a good mastery of implementation method of the TBHE patterns.

#### WeChat mini-program development

The WeChat mini-program for this project was completed by Android software engineers of Japan Forest Software Co., Ltd. (フレストソフト Co., Ltd.) and their team. The system adopted a Client-Server(C/S) structure, and its master functions included four modules: health education learning, learning record, practice, and evaluation. The server was mainly used to manage and count user information data, which could perform the release of health education videos, count the number of users, practice scores, and evaluation scores, and display them to managers in the form of pie charts and line charts.

#### Intervention process

The same health education content was provided to both the intervention and control groups following eight themes related to falls: physiology, disease drugs, mental health, cognition, lifestyle, behavior, and general and particular environment. Intervention frequency was the same for both groups. At the end of the weekly health education, the intervenor paid the same remuneration to the two groups of participants to ensure that they continue participating in this study in the WeChat group and to express our gratitude. The randomized controlled trial protocol is shown in Table 1.

**Table 1:** Overview of the intervention process

		Intervention group (*n* = 30)	Control group (*n* = 30)
**Participants**		Older adults aged 60 and above who could use WeChat	Older adults aged 60 and above who could use WeChat
**Health education content**		According to results of previous Fall Prevention at Home Knowledge (FPHK) questionnaire, we developed 8 health education videos related to falls, including physiology, disease drugs, mental health, cognition, lifestyle, behavior, general and particular environment. The length of each video ranges from 5 to 26 minutes.	According to results of previous Fall Prevention at Home Knowledge (FPHK) questionnaire, we developed 8 health education videos related to falls, including physiology, disease drugs, mental health, cognition, lifestyle, behavior, general and particular environment. The length of each video ranges from 5 to 26 minutes.
**Intervention frequency**		Every Tuesday and Friday, starting at 8:00 a.m., for eight consecutive weeks, twice a week	Every Tuesday and Friday, starting at 8:00 a.m., for eight consecutive weeks, twice a week
**Intervention time**		Intervention time for each person on Tuesday was 20-30 minutes, and Friday was 60-90 minutes.	Intervention time for each person on Tuesday was 20-30 minutes, and Friday was 20-30 minutes.
**Intervention measure**		Teach-back health education (TBHE)	Traditional health education
**Specific health education process**	Tuesday 8 a.m.	The first author sent WeChat mini-programs code to WeChat group. Participants scanned the code and entered WeChat mini-programs to watch and learn videos which fall prevention at home health education contents	The first author sent WeChat mini-programs code to WeChat group. Participants scanned the code and entered WeChat mini-programs to watch and learn videos which fall prevention at home health education contents.
	Friday 8 a.m.	Participants entered WeChat mini-programs again to learn eight topics content on preventing falls at home and evaluate satisfaction. The first author applied a cycle of assessment, education, reassessment, and reeducation to conduct teach-back health education.	Participants entered WeChat mini-programs again to learn eight topics content on preventing falls at home and evaluate satisfaction. What participants learned on Friday was the same as on Tuesday.
**Instruments**	Before intervention	a. Socio-demographic questionnaire b.The FPHK questionnaire	a. Socio-demographic questionnaire b.The FPHK questionnaire
	After intervention	The FPHK questionnaire	The FPHK questionnaire

#### Intervention group

The TBHE intervention was used for participants in the intervention group and was achieved through a spiral cycle of assessment-education-reassessment-reeducation. Taking the lifestyle educational theme as an example, its content includes three video animations of Baduanjin, Tai Chi, and Five-Animal Exercises and two text descriptions about yoga and jogging. The purpose is to guide older adults to be aware of the importance of developing a good exercise lifestyle, forming a good living habit of daily exercise, and avoiding falls at home. Considering the Baduanjin or Eight-Section Brocades as an example, it consists of eight individual movements and is one of the traditional Chinese Qigong exercise therapies characterized by symmetrical body postures and movements, breathing control, a meditative state of mind, and mental focus [25]. Baduanjin could effectively improve balance, leg muscle strength, mobility, and fall prevention [26]. The TBHE intervention process was as follows: (a) The gaps in theoretical knowledge and practical skills in the fall-related lifestyle of older adults was assessed. FA informed participants that they needed to enter the WeChat mini-program every Tuesday to watch 5 min and 14 s of lifestyle animation videos and complete five practice questions related to lifestyle. After completing the questions, FA immediately reported errors to participants. According to a standard answer, FA explained the correct content to the participants and rightly guided them. Participants needed to record a video of themselves completing Baduanjin before the coming Friday and send it to FA in advance through WeChat. FA combined the participants’ previous practice questions and recorded exercise videos to manually analyze whether the participants fully mastered the theoretical knowledge and sports skills according to the correct answers and standard exercise movements. Finally, FA scanned and evaluated the total distribution of wrong questions and each person’s sports practices in the WeChat mini-program background, which recorded weak points in each person’s theory and practice. (b) Feedback on weak points in theory and practice were given to the older adults, and they were educated to re-examine gaps to improve. FA asked the participants to watch the Baduanjin animation video on Friday and complete the same five exercises again. Then, FA corrected the weak points of each person’s theoretical knowledge and practical skills one-to-one through pictures, voice, and videos. Participants recited the health education content in their language in voice format, gave feedback on sports skills to be mastered in video format, and sent them to FA again. (c) Re-evaluation and re-education: FA re-evaluated participants’ retelling voice and sports video, corrected them again according to the unified standard, and repeated the above process. The TBHE intervention ended only when the participants could correctly repeat all the right knowledge points taught to them during the health education and complete the whole set of Baduanjin movements skillfully and standardly, which indicated that they mastered the theoretical knowledge and sports skills. Finally, participants rated their satisfaction after learning the entire health education content on the topic of lifestyle on Friday.

#### Control group

Traditional health education was used for participants in the control group. Participants were reminded to enter the WeChat mini-program at 8 am every Tuesday to learn health education content on the topic of preventing falls at home and completed the same five exercises as the intervention group. During this research, FA did not perform any active interventions and only responded to participants who had questions, which was also ethical. On Friday, FA urged them to re-enter the WeChat mini-program to learn the health education content on fall prevention at home consistent with the theme of Tuesday. Finally, participants rated their satisfaction after learning the entire health education content on the topic of lifestyle on Friday. After 8 weeks, participants finished learning eight topics content, and traditional health education ended.

### Instruments

#### Socio-demographic questionnaire

A self-designed questionnaire was used to collect sociodemographic information about older adults. Older adults’ sociodemographic data included age, gender, marital status, children number, education, having chronic diseases or not, living with children or not, public officials or not, primary caregiver, monthly income, self-care ability, and smoking, drinking, and exercise frequency.

#### Home-based fall prevention knowledge (HFPK) questionnaire

The HFPK was developed based on the Health Belief Model (HBM) [27], Expert Consensus on Fall Risk Assessment of Older Adults in China (draft) [28], and Fall Prevention Knowledge, Attitude, and Practice Questionnaire [29, 30]. The HFPK assessed urban older adults’ knowledge of fall prevention, including physiological diseases, application of drugs, psychological cognition and mental status, lifestyle and behavior, and knowledge of the house environment that older adults should be aware of to prevent falls. We emailed 15 community or aged care specialists for comments on the initial questionnaire using Delphi Method. After two rounds of consultation and revision of the questionnaire, we tested the reliability and validity of the HFPK among 374 community-based older adults aged 60 years and above in Fuzhou. The formal HFPK comprises 68 items grouped into five subscales, including physiology & disease (19 items, 0 ~ 19 scores), drug application (8 items, 0 ~ 8 scores), mental, cognitive, and spiritual well-being (12 items, 0 ~ 12 scores), lifestyle (8 items, 0 ~ 8 scores), and house environment (21 items, 0 ~ 21 scores). Its score ranges from 0 to 68. All items are positive, with three answers including yes, uncertain, and no, which are all multiple-choice questions. If answer is Yes, the response is correct and gets 1 point. If answer is unclear or no, the response is incorrect and gets 0. The higher the score, the higher the knowledge level of older adults in preventing falls at home. According to the 100-point scale, 85 ~ 100 is excellent, 75 ~ 84 is good, 60 ~ 74 is medium, and 60 below is poor [31]. Knowledge on preventing falls at home was categorized as follows: 58 ~ 68 as excellent, 51 ~ 57 as good, 41 ~ 50 as medium, and 40 below as poor. After expert evaluations, I-CVI ranged from 0.867 to 1, and S-CVI was 0.985, which achieved the content validity criterion [32], showing that questionnaire items were accurate and comprehensive. Cronbach’α coefficient was 0.953, which met the reliability criterion, indicating that what is measured tends to be more consistent [33].

### Satisfaction and feedback

Older adults who completed each learning on Friday were asked to enter the user evaluation module of the WeChat mini-program to evaluate their satisfaction with the health education content, form, and content applicability separately on a 5-point Likert scale (1 = very dissatisfied, 5 = very satisfied). Each person performed 8 health education and 8 satisfaction evaluations. Finally, two questions were used to collect participants’ feedback on the TBHE program: ‘Were there any questions disturbing you during the interview?’ and ‘Would you recommend the TBHE to other older adults?’

### Data collection and statistical analysis

Research assistants who were blinded to the assignment of groups during collected data. Participants were surveyed face-to-face using questionnaires before and 1 week after the intervention. Data were analyzed using IBM SPSS Statistics for Windows, version 25.0 (IBM SPSS Data Collection, New York, NY, USA). Mann-Whitney U test and chi-square were used to compare the baseline data of two the two groups. When there was a difference in baseline data between the two groups, covariance tests were used. A missing case in the intervention group was excluded from the analysis. A two-way repeated-measures analysis of variance (ANOVA) was used to explore between-group (group: intervention vs. control), within-group (time: baseline and after 1 week), and interaction (group∗time) effects. Criterion for statistical significance was set at *p* < 0.05 in a two-tailed test. An independent-samples t-test was used for pairwise comparisons between two groups, and a paired-samples t-test was used to compare outcomes at different time points within groups.

### Ethical considerations

This study was approved by the Ethics Committee of the Fujian Medical University (IRB Ref. No.: 2020/00,081). Participants were provided with detailed information about the study and written informed consent was obtained from each participant prior to data collection. Importantly, all questionnaire information could not be shown to anyone else except researchers without their consent.

## Results

### Demographics

A total of 60 participants thus agreed to participate in this study and were randomly assigned to the intervention group (*n* = 30) or the control group (*n* = 30). One participant in the intervention group withdrew from our study at the first week after the intervention because his spouse was hospitalized and needed his company and care. The drop-out rate of this study was 1.67%. Finally, 29 participants in the intervention group and 30 ones in the control group were included in the outcome analysis. The flow chart for recruitment and specific reasons for participants’ drop-out are shown in Fig. 1. There was no significant difference in baseline data between the two groups (*p* > 0.05), and the groups were balanced and comparable as shown in Table 2.

**Table 2:** Baseline data comparison results between the intervention group and the control group

Value		Intervention group (*n* = 29)		Control group (*n* = 30)		Statistics	*P*-value
		*n*	*%*	*n*	*%*		
**Gender**	Male	16	55.17	14	46.67	0.427 ^a^	0.514
	Female	13	44.83	16	53.33		
**Age**	60 ~ 69	19	65.52	26	86.67	3.644 ^a^	0.056
	≥70	10	34.48	4	13.33		
**Marital status**	Married	28	96.55	30	100	1.052 ^a^	0.492
	No spouse	1	3.45	0	0		
**Children number**	0 ~ 1	11	37.93	13	43.33	0.178 ^a^	0.792
	≥2	18	62.07	17	56.67		
**Education**	Illiteracy	7	24.14	6	20.00	−0.109^b^	0.913
	Primary school	0	0	1	3.33		
	Junior high school	10	34.48	7	23.33		
	High school or secondary school	4	13.79	10	33.33		
	College	3	10.34	3	10.00		
	Undergraduate and above	5	17.24	3	10.00		
**Suffering from chronic diseases**	Yes	13	44.83	16	53.33	0.427 ^a^	0.606
	No	16	55.17	14	46.67		
**Living with children**	Yes	17	58.62	20	66.67	0.408 ^a^	0.596
	No	12	41.38	10	33.33		
**Primary caregiver**	Self-care	19	65.52	21	70.00	0.136 ^a^	0.785
	No self-care	10	34.48	9	30.00		
**Public officials**	Yes	4	13.79	13	43.33	5.519 ^c^	0.009
	No	25	86.20	17	56.67		
**Monthly income**	<1000	6	20.69	2	6.67	−0.008^b^	0.994
	1000 ~ 1999	2	6.90	5	16.67		
	2000 ~ 3999	10	34.48	14	46.67		
	≥4000	11	37.93	9	30.00		
**Self-care ability**	Fully self-care	28	96.55	30	100	1.052 ^a^	0.492
	Not fully self-sufficient	1	3.45	0	0		
**Smoking frequency**	Never	26	89.65	28	93.33	−0.518^b^	0.604
	1-2 days a week	1	3.45	1	3.33		
	3-4 days a week	0	0	0	0		
	Every day	2	6.90	1	3.33		
**Drinking frequency**	Never	25	86.20	24	80.00	−0.592^b^	0.554
	1-2 days a week	1	3.45	5	16.67		
	3-4 days a week	1	3.45	0	0		
	Every day	2	6.90	1	3.33		
**Exercise frequency**	Never	5	17.24	10	33.33	−1.753^b^	0.080
	1-2 days a week	2	6.90	3	10.00		
	3-4 days a week	1	3.45	2	6.67		
	Every day	21	72.41	15	50.00		

^a^The chi-square test is used, and the statistic is *χ*^*2*^-value

^b^The rank sum test is used, and the statistic is the *Z*-value

^c^The covariance analysis is used, and the statistic is the *F*-value

### Comparison of outcome variables of older adults between intervention and control groups at different time points

**Fig. 2:**
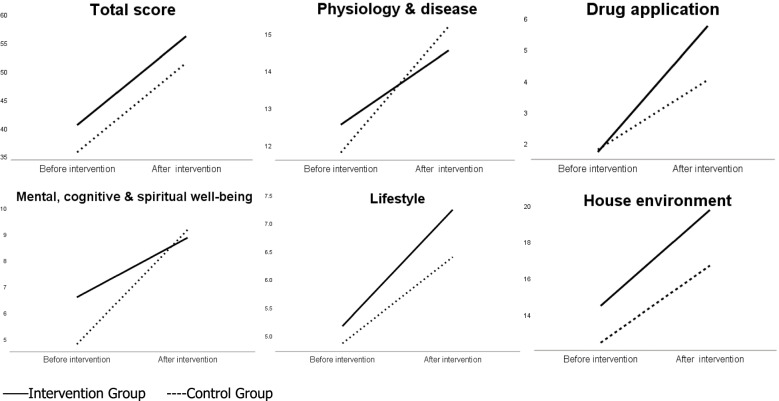
Profile plots of interaction effects before and after the intervention of total score and five dimensions scores

Table 3 shows the comparison of outcome variables of older adults between the intervention and control groups at different time points. Profile plots of interaction effects before and after the intervention are shown in Fig. 2. There was no significant difference in total score (*t* = 1.433, *p* = 0.157), physiology and disease (*t* = 0.667, *p* = 0.508), drug application (*t* = − 0.135, *p* = 0.893), lifestyle (*t* = 0.677, *p* = 0.501), and house environment (*t* = 1.663, *p* = 0.102) scores between the intervention and control groups at T0, except for mental, cognitive, and spiritual well-being (*t* = 2.410, *p* = 0.019). Meanwhile, results of comparison between the two groups showed that older adults in the intervention group had a higher total score (*t* = 2.194, *p* = 0.032), drug application (*t* = 2.967, *p* = 0.004), lifestyle (*t* = 2.653, *p* = 0.010), and house environment (*t* = 4.575, *p* < 0.001) scores at T1, indicating that older adults’ scores for total situation, drug application, lifestyle, and house environment in the intervention group were generally higher than those in the control group. The interaction effects were significant for drug application (*F* = 6.811, *p* = 0.012) and mental, cognitive, and spiritual well-being (*F* = 6.262, *p* = 0.015), indicating that these outcomes of participants were affected by the time and intervention. After the intervention, scores for drug application in the intervention group were higher than those in the control group. Interestingly, after the intervention, scores for mental, cognitive, and spiritual well-being in the control group were higher than in the intervention group. In addition, although the interaction effect had no significant difference (*p* > 0.05) in total score, physiology and disease, lifestyle, and home environment, the scores for the total situation (*t* = 2.194, *p* = 0.032), lifestyle (*t* = 2.653, *p* = 0.010), and house environment (*t* = 4.575, *p* < 0.001) of the intervention group were higher than the control group after the intervention.

**Table 3:** Comparison of outcome variables of older adults between intervention and control groups at different time points

	Dimensions	Group (*n*)	T0 (Mean ± SD)	T1 (Mean ± SD)	Between groups F(*p*)	Within group F(*p*)	Interaction effect F(*p*)
**ANOVA analysis**^a^	Total score	Intervention (29)	40.52 ± 14.20	56.17 ± 8.13	3.554 (0.065)	159.714 (< 0.001)	0.002 (0.964)
	(range of scores:0 ~ 68)	Control (30)	35.73 ± 11.31	51.50 ± 8.22			
	Physiology and disease	Intervention (29)	12.55 ± 4.63	14.55 ± 2.84	0.004 (0.949)	31.214 (< 0.001)	2.098 (0.153)
	(range of scores:0 ~ 19)	Control (30)	11.80 ± 4.02	15.20 ± 2.67			
	Drug application	Intervention (29)	1.72 ± 2.25	5.76 ± 2.15	3.279 (0.075)	82.481 (< 0.001)	6.811 (0.012)
	(range of scores:0 ~ 8)	Control (30)	1.80 ± 2.06	4.03 ± 2.31			
	Mental, cognitive, and spiritual well-being	Intervention (29)	6.59 ± 3.21	8.86 ± 2.39	2.014 (0.161)	63.205 (< 0.001)	6.262 (0.015)
	(range of scores:0 ~ 12)	Control (30)	4.80 ± 2.44	9.17 ± 2.12			
	Lifestyle	Intervention (29)	5.17 ± 1.83	7.24 ± 0.91	3.152 (0.081)	67.607 (< 0.001)	1.495 (0.227)
	(range of scores:0 ~ 8)	Control (30)	4.87 ± 1.63	6.40 ± 1.45			
	House environment	Intervention (29)	14.48 ± 4.86	19.76 ± 1.96	9.381 (0.003)	83.231 (< 0.001)	1.000 (0.321)
	(range of scores:0 ~ 21)	Control (30)	12.47 ± 4.45	16.70 ± 3.04			
	**Dimensions**	**Group (*n*)**	**T0 (*t*,*p*)**	**T1 (*t*,*p*)**			
**Comparison between groups**^b^	Total score	Intervention (29)	1.433, 0.157	2.194, 0.032			
		Control (30)					
	Physiology and disease	Intervention (29)	0.667, 0.508	−0.904, 0.370			
		Control (30)					
	Drug application	Intervention (29)	−0.135, 0.893	2.967, 0.004			
		Control (30)					
	Mental, cognitive, and spiritual well-being	Intervention (29)	2.410, 0.019	−0.519, 0.606			
		Control (30)					
	Lifestyle	Intervention (29)	0.677, 0.501	2.653, 0.010			
		Control (30)					
	House environment	Intervention (29)	1.663, 0.102	4.575, < 0.001			
		Control (30)					
	**Dimensions**	**Group (n)**	**T0 vs T1 (*t*,*p*)**				
**Comparison within groups**^c^	Total score	Intervention (29)	−8.344, < 0.001				
		Control (30)	−9.619, < 0.001				
	Physiology and disease	Intervention (29)	−2.857, 0.008				
		Control (30)	−5.096, < 0.001				
	Drug application	Intervention (29)	−8.499, < 0.001				
		Control (30)	−4.468, < 0.001				
	Mental, cognitive, and spiritual well-being	Intervention (29)	−3.448, 0.002				
		Control (30)	−8.438, < 0.001				
	Lifestyle	Intervention (29)	−6.955, < 0.001				
		Control (30)	−4.782, < 0.001				
	House environment	Intervention (29)	−7.202, < 0.001				
		Control (30)	−5.715, < 0.001				

*SD* Standard deviation

^a^The outcomes were based on two-way analysis of variance (ANOVA) of repeated measures

^b^The outcomes were analysed using independent-samples T test

^c^The outcomes were analysed using paired-samples T test

### Satisfaction and feedback

The study showed that all participants provided positive evaluations (very satisfactory and relatively satisfactory) of the TBHE intervention regarding the health education content and content applicability. In addition, the majority of older adults (78.81%) provided positive evaluations (very satisfactory and relatively satisfactory) of the TBHE intervention in the health education form. No participants were unsatisfied with the intervention. Moreover, participants were willing to recommend the TBHE program to other older adults. The intervention frequency set at the beginning was 2 days apart, and participants reported that the frequency was fast. FA therefore extended the weekly intervention interval.

### Compliance

Although participating older adults were generally satisfied with the content, form, and content applicability of TBHE, participants’ adherence was poor at the beginning of this study. The first author sent the program code of the WeChat mini-program at 8:00 in the morning to remind participants to join the WeChat mini-program to watch videos about fall prevention. However, the participants did not watch videos immediately, and some of them did not finish watching them until 10 p.m. The first author needed to contact them and send voice reminders multiple times. Later, she patiently communicated with the participants about the significance of this study in promoting their fall-prevention cognition and regularly distributed monetary rewards to each participant in the WeChat group. Therefore, the participant compliance was gradually improved.

## Discussion

TBHE provides real-time feedback on the health education effect that could increase older adults’ fall prevention knowledge about drug application compared to a traditional form that health promoters directly one-way transfer lore or skills to recipients. Moreover, older adults in the intervention group also had better fall-prevention knowledge scores regarding their total condition, lifestyle, and house environment than those in the control group, indicating the efficiency of TBHE in improving the above fall prevention knowledge scores. Interestingly, we found that after the intervention, mental, cognitive, and spiritual well-being scores were higher in the control group than in the intervention group. We reasonably and optimistically conjecture that the intervention in the control group would also be beneficial in improving mental, cognitive, and spiritual well-being knowledge in older adults. Nonetheless, it is undeniable that TBHE still has many advantages. First, unlike establishing WeChat groups for health education [34], participants used the WeChat mini-program developed by our team is convenient to take health education without downloading other applications to occupy the storage of phones and prevent users from being disturbed by other strangers. Second, the one-to-one approach makes participants feel more focused, thus encouraging them to absorb knowledge fully. Third, some were satisfied with this novel and accessible health education tool and willing to recommend it to others to improve their fall prevention knowledge. Of course, not all older adults were willing to accept this program, which indicates that it is suitable and effective only for those participants who would join it and devote themselves to it.

In drug application, our intervention work has two contributions to modern-day studies on fall prevention in older adults. First, in previous research on health education interventions to prevent falls, few studies were conducted on the drug knowledge of older adults alone and only investigated whether older adults know some drugs that are likely to cause falls among them. Moreover, present studies surveyed drug types incomprehensively. For example, Quan [35] only listed sleeping pills, sedatives, hypotension, hypoglycemic agents, and diuretics. Our study listed as many drug types which hoped older adults could know related to falling as possible based on the literature review, and many relevant experts unanimously recognized them. Second, Aiping [36] conducted a seven-month teach-back combined with video health education intervention on pulmonary inhalant usage in 163 COPD patients. She found that the teach-back health education could significantly improve participants’ knowledge of pulmonary inhalers usage in the intervention group. Likewise, in our study, a higher knowledge score in the intervention group further confirms the effectiveness of TBHE in drug application knowledge. Before the intervention, two groups had low scores with fall-prevention knowledge regarding their drug application knowledge, which was consistent with the finding of Liping [37] that the awareness rate of drug application knowledge among older adults was lower than 30%. After the intervention, the drug application knowledge score of the intervention group was significantly improved and better than that of the control group. Factually, the meaning of receiving teach-back health education could remind older adults to pay attention to the relationship between drugs and falls when taking various medicines daily so that they are more careful and correct to take pills as directed by their doctors.

In terms of mental, cognitive, and spiritual well-being, the incidence of mental illness, like depression, is increasing year in the elderly population [38]. Compared to other clinical diseases in older adults, depression may be underdiagnosed. People with depression are less likely to seek therapy due to the stigma of mental illness in some areas of China [39]. This study makes older adults realize that they need to attach importance to their mental state by educating them that abnormal psychological states such as emotional stress can easily lead to falls. An interesting finding of this study is that, despite the interaction of time and group on mental, cognitive, and spiritual well-being, the control group scored higher than the intervention group after the intervention. There are two possible reasons as follows. First, the baseline data for mental, cognitive, and spiritual well-being are unbalanced. Before the intervention, the mental, cognitive, and spiritual well-being scores of the intervention group were higher than those of the control group, and the difference was statistically significant (*t* = 2.410, *p* = 0.019). After the intervention, the score increase of the intervention group was lower than that of the control group. Second, the intervention in the control group affected the participants. The interventions in the control group were self-learning, and the FA did not engage in any active interventions and responded only to problematic participants. In the satisfaction and feedback, participants mentioned that they thanked the FA for spending time to help them improve their knowledge, patiently answering their questions, and soothing their emotions when they encountered WeChat use obstacles. These measures stimulated their interest in learning and maintained it until the end of the study, creating conditions for participants to learn knowledge, consistent with a comparison within groups results. Self-contrast found that two groups in mental, cognitive, and spiritual well-being were statistically significant (*t* = − 3.448, *p* = 0.002; *t* = − 8.438, *p* < 0.001), showing that TBHE and traditional health education intervention could increase scores in this dimension. Thus, our project helps to remind participants to value their mental health, maintain a peaceful mind, actively seek psychological treatment, and prevent falls.

In terms of lifestyle and the household environment, participants in the intervention group had significantly higher knowledge scores for fall prevention than those in the control group; however, there was no interaction effect. Zhihong [40] pointed out that a restricted household environment and an unhealthy lifestyle were predisposing factors for falls in older adults and that reducing the per capita housing area and drinking alcohol would increase the incidence of falls. In addition, technical guidelines for fall interventions in older adults [41] pointed out that it is necessary to improve living environments to be suitable for older adults to prevent home-based injuries. Our study suggests that TBHE reinforces older adults’ fall prevention knowledge about their lifestyle and household environment and reminds them to keep healthy living habits and improve their household environment. It is worth mentioning that there was no significant difference in physiology and disease (*t* = − 0.904, *p* = 0.370). A study [42] showed that older adults were aware of their decreased physical performance and reduced ability to cope with physical challenges, such as balance, which increased their fear of falling. Fear of falling may cause them to seek out a lot of fall prevention knowledge about physical health. Therefore, learning fall prevention knowledge in physiology and disease is still significant.

### Limitations

This study has some limitations. First, the implementation and evaluation process of TBHE were all completed in WeChat without having to meet the participants face-to-face. The authenticity of feedback from participants could not be guaranteed, which was likely to affect the quality of TBHE. Second, we do have not a larger study sample, a higher intervention frequency, and a longer study duration. Scholars in the future can consider increasing the size of the study sample and extending the intervention’s duration to better explore the effect of the intervention. Finally, the WeChat mini-program could only be used on smartphones, which hinders some older adults who use outdated phones from benefiting from such health education. Future researchers need to design more rational health education programs for older adults who do not use smartphones.

## Supplementary Information

**Additional file 1.**

## Data Availability

The datasets generated and/or analyzed during the current study are not publicly available due this article is part of the first author’s master’s thesis within two years of confidentiality. The datasets are not suitable for publication now but are available from the corresponding author on reasonable request.
