# Supplementary material for: Effects of teach-back health education (TBHE) based on WeChat mini-programs in preventing falls at home for urban older adults in China: a randomized controlled trial
Source: BMC Geriatr. 2022 Jul 23;22:611. doi: 10.1186/s12877-022-03297-9 (PMC9308328; doi:10.1186/s12877-022-03297-9)
Supplement: Supplementary file 1 — Additional file 1. [file 12877_2022_3297_MOESM1_ESM.docx]

**Effects of teach-back health education (TBHE) based on WeChat mini-programs in preventing falls at home for urban older adults: A randomized controlled trial**

**Appendix - Home-based Fall Prevention Knowledge (HFPK) questionnaire**

**Guidance:** We are conducting a study on Home-based Fall Prevention Knowledge (HFPK) questionnaire. Falls are the primary cause of accidental injuries among the elderly. To understand the present situation which falls prevention knowledge for urban older adults, we have designed a questionnaire on knowledge about fall prevention at home for urban older adults concerning the relevant literature. Please tick "√" in the column you think is appropriate.

| Items | Yes | Uncertainty | No |
| --- | --- | --- | --- |
| 1. Aging increases the risk of falls.  1.年龄的增长会增加跌倒的风险。 | **□** | **□** | **□** |
| 2. Vision loss increases the risk of falls.  2.视力减退会增加跌倒的风险。 | □ | □ | □ |
| 3. Hearing loss can increase the risk of falls.  3.听觉减退会增加跌倒的风险。 | □ | □ | □ |
| 4. Decreased sense of touch can increase the risk of falls.  4.触觉减退会增加跌倒的风险。 | □ | □ | □ |
| 5. Decreased balance increases the risk of falls.  5.平衡能力下降会增加跌倒的风险。 | □ | □ | □ |
| 6. Reduced muscle strength in the lower extremities increases the risk of falls.  6.下肢肌力减退会增加跌倒的风险。 | □ | □ | □ |
| 7. Syncope increases the risk of falls.  7.晕厥会增加跌倒的风险。 | **□** | **□** | **□** |
| 8. Pain episodes can increase the risk of falls.  8.疼痛发作会增加跌倒的风险。 | □ | □ | □ |
| 9. Poor sleep quality can increase the risk of falls.  9.睡眠质量不好会增加跌倒的风险。 | □ | □ | □ |
| 10. Epilepsy can increase the risk of falls.  10.癫痫会增加跌倒的风险。 | □ | □ | □ |
| 11. Parkinson's disease can increase the risk of falls.  11.帕金森病会增加跌倒的风险。 | □ | □ | □ |
| 12. Alzheimer's increases the risk of falls.  12.老年痴呆会增加跌倒的风险。 | □ | □ | □ |
| 13. Stroke increases the risk of falls.  13.脑卒中会增加跌倒的风险。 | **□** | **□** | **□** |
| 14. Hypotension increases the risk of falls.  14.低血压会增加跌倒的风险。 | □ | □ | □ |
| 15. Hypertension can increase the risk of falls.  15.高血压会增加跌倒的风险。 | □ | □ | □ |
| 16. Arrhythmias increase the risk of falls.  16.心律失常会增加跌倒的风险。 | □ | □ | □ |
| 17. Osteoarthritis can increase the risk of falls.  17.骨关节炎会增加跌倒的风险。 | □ | □ | □ |
| 18. Osteoporosis can increase the risk of falls.  18.骨质疏松会增加跌倒风险。 | □ | □ | □ |
| 19. Cervical spondylosis increases the risk of falls.  19.颈椎病会增加跌倒风险。 | **□** | **□** | **□** |
| 20. The use of psychotropic drugs increases the risk of falls.  20.使用精神类药会增加跌倒的风险。 | □ | □ | □ |
| 21. The use of sedative pills increases the risk of falls.  21.使用镇静安眠类药会增加跌倒的风险。 | □ | □ | □ |
| 22. The use of cardiovascular drugs increases the risk of falls.  22.使用心血管药会增加跌倒的风险。 | □ | □ | □ |
| 23. The use of antiarrhythmic drugs increases the risk of falls.  23.使用抗心律失常药会增加跌倒的风险。 | □ | □ | □ |
| 24. The use of antiepileptic drugs increases the risk of falls.  24.使用抗癫痫药会增加跌倒的风险。 | □ | □ | □ |
| 25. Anti-dizziness drugs increase the risk of falls.  25.抗晕动病药会增加跌倒的风险。 | **□** | **□** | **□** |
| 26. The use of hypoglycemic drugs increases the risk of falls.  26.使用降糖药会增加跌倒的风险。 | □ | □ | □ |
| 27. The use of central analgesics increases the risk of falls.  27.使用中枢性镇痛药会增加跌倒的风险。 | □ | □ | □ |
| 28. Depression can increase the risk of falls.  28.抑郁症会增加跌倒的风险。 | □ | □ | □ |
| 29. Anxiety disorders can increase the risk of falls.  29.焦虑症会增加跌倒的风险。 | □ | □ | □ |
| 30. Mania can increase the risk of falls.  30.狂躁症会增加跌倒的风险。 | □ | □ | □ |
| 31. Phobias can increase the risk of falls.  31.恐惧症会增加跌倒的风险。 | □ | □ | □ |
| 32.Obsessive-compulsive disorder (OCD) can increase the risk of falls.  32.强迫症会增加跌倒的风险。 | □ | □ | □ |
| 33. Schizophrenia increases the risk of falls.  33.精神分裂症会增加跌倒的风险。 | □ | □ | □ |
| 34. Low or impaired cognition can increase the risk of falls.  34.认知低下或障碍会增加跌倒的风险。 | □ | □ | □ |
| 35. Overestimation of your condition increases the risk of falls.  35.对自身状况的高估会增加跌倒的风险。 | □ | □ | □ |
| 36. Insufficient awareness of fall risk increases the risk of falls.  36.对跌倒风险认识不足会增加跌倒的风险。 | □ | □ | □ |
| 37. Decreased responsiveness increases the risk of falls.  37.反应敏捷度下降会增加跌倒的风险。 | □ | □ | □ |
| 38. Fear of falling increases the risk of falling.  38.害怕跌倒会增加跌倒的风险。 | □ | □ | □ |
| 39. A history of falls within 6 months increases the risk of falls.  39.6个月内有跌倒史会增加跌倒的风险。 | □ | □ | □ |
| 40. Long clothes and pants can increase the risk of falls.  40.衣服裤子过长会增加跌倒的风险。 | □ | □ | □ |
| 41. Improperly sized or non-slip shoes can increase the risk of falls.  41.鞋子大小不合适或不防滑会增加跌倒的风险。 | □ | □ | □ |
| 42. Wearing too much clothing can increase the risk of falls.   1. 衣物穿着过多会增加跌倒的风险。 | □ | □ | □ |
| 43. A sudden change of position increases the risk of falls.  43.突然改变体位会增加跌倒的风险。 | □ | □ | □ |
| 44. Climbing for objects can increase the risk of falling.  44.登高取物会增加跌倒的风险。 | □ | □ | □ |
| 45. Lifting heavy objects increases the risk of falling.  45.提重物会增加跌倒的风险。 | □ | □ | □ |
| 46. Getting up frequently at night increases the risk of falls.  46.夜间频繁起夜会增加跌倒的风险。 | □ | □ | □ |
| 47. Exercise increases the risk of falls.  47.运动锻炼会增加跌倒的风险。 | □ | □ | □ |
| 48. Poor indoor lighting can increase the risk of falls.  48.室内光线不良会增加跌倒的风险。 | □ | □ | □ |
| 49. Poor indoor ventilation can increase the risk of falls.  49.室内通风不良会增加跌倒的风险。 | □ | □ | □ |
| 50. A slippery indoor floor increases the risk of falls.  50.室内地面湿滑会增加跌倒的风险。 | □ | □ | □ |
| 51. Small indoor spaces can increase the risk of falls.  51.室内空间狭小会增加跌倒的风险。 | □ | □ | □ |
| 52. Cluttered indoor objects can increase the risk of falls.  52.室内物品摆放杂乱会增加跌倒的风险。 | □ | □ | □ |
| 53. The lack of handrails in indoor activity venues can increase the risk of falls.  53.室内活动场所缺乏扶手会增加跌倒的风险。 | □ | □ | □ |
| 54. High indoor stairs can increase the risk of falls.  54.室内楼梯阶层过高会增加跌倒的风险。 | □ | □ | □ |
| 55. The lack of fall prevention signs on indoor stair steps can increase the risk of falls.  55.室内楼梯台阶缺乏防跌标识会增加跌倒的风险。 | □ | □ | □ |
| 56. The turning corner of indoor stairs increases the risk of falls.  56. 室内楼梯转角过大会增加跌倒的风险。 | □ | □ | □ |
| 57. Lack of right-angle protectors at the edges or corners of indoor furniture can increase the risk of falls.  57.室内家具边缘或转角处缺乏直角保护器会增加跌倒的风险。 | □ | □ | □ |
| 58. Uneven indoor floors can increase the risk of falls.  58.室内地面不平整会增加跌倒的风险。 | □ | □ | □ |
| 59. High interior door thresholds increase the risk of falls.  59.室内门槛过高会增加跌倒的风险。 | □ | □ | □ |
| 60. The height of the bed, seat, and sofa is too low or too high will increase the risk of falling.  60.床铺、座椅、沙发高度过低或过高会增加跌倒的风险。 | □ | □ | □ |
| 61. Kitchen without non-slip tiles or non-slip mats will increase the risk of falls.  61.厨房未铺防滑砖或防滑垫会增加跌倒的风险。 | □ | □ | □ |
| 62. Too much debris on the balcony can increase the risk of falls.  62.阳台杂物过多会增加跌倒的风险。 | □ | □ | □ |
| 63. The lack of non-slip mats on the bathroom floor increases the risk of falls.  63.卫生间地面缺乏防滑垫会增加跌倒的风险。 | □ | □ | □ |
| 64. Lack of handrails next to the bathroom or toilet increases the risk of falls.  64.浴室或马桶旁缺乏扶手会增加跌倒的风险。 | □ | □ | □ |
| 65. Using a bathtub increases the risk of falls.  65.使用浴缸会增加跌倒的风险。 | □ | □ | □ |
| 66. Lack of seats or weak seats in the shower increases the risk of falls.  66.淋浴间缺乏座椅或座椅不牢固会增加跌倒的风险。 | □ | □ | □ |
| 67. Non-slip slippers in the shower increases the risk of falls.  67.淋浴时未穿防滑拖鞋会增加跌倒的风险。 | □ | □ | □ |
| 68. Dry and wet toilet without separation can increase the risk of falls.  68.卫生间干湿未分离会增加跌倒的风险。 | □ | □ | □ |
